# Quality of reporting internal and external validity data from randomized controlled trials evaluating stents for percutaneous coronary intervention

**DOI:** 10.1186/1471-2288-9-24

**Published:** 2009-04-09

**Authors:** Morgane Ethgen, lsabelle Boutron, Philippe Gabriel Steg, Carine Roy, Philippe Ravaud

**Affiliations:** 1 Institut National de la Santé et la Recherche Médiale, (INSERM U738), Paris, France; Université Paris Diderot Paris 7, UFR de Médecine, Paris, France; AP-HP, Hôpital Bichat, Département d'Epidémiologie, Biostatistique et Recherche Clinique, Paris, France; 2 Université Paris Diderot Paris 7, UFR de Médecine, Paris, France; AP-HP, Hôpital Bichat, and INSERM U-698, Paris, France

## Abstract

**Background:**

Stents are commonly used to treat patients with coronary artery disease. However, the quality of reporting internal and external validity data in published reports of randomised controlled trials (RCTs) of stents has never been assessed.

The objective of our study was to evaluate the quality of reporting internal and external validity data in published reports of RCTs assessing the stents for percutaneous coronary interventions.

**Methods:**

A systematic literature review was conducted. Reports of RCTs assessing stents for percutaneous coronary interventions indexed in MEDLINE and the Cochrane Central Register of Controlled Trials and published between January 2003 and September 2008 were selected. A standardized abstraction form was used to extract data. All analyses were adjusted for the effect of clustering articles by journal.

**Results:**

132 articles were analyzed. The generation of the allocation sequence was adequate in 58.3% of the reports; treatment allocation was concealed in 34.8%. Adequate blinding was reported in one-fifth of the reports. An intention-to-treat analysis was described in 79.5%. The main outcome was a surrogate angiographic endpoint in 47.0%. The volume of interventions per center was described in two reports. Operator expertise was described in five (3.8%) reports. The quality of reporting was better in journals with high impact factors and in journals endorsing the CONSORT statement.

**Conclusion:**

The current reporting of results of RCTs testing stents needs to be improved to allow readers to appraise the risk of bias and the applicability of the results.

## Background

In the past decade, stenting has become a routine treatment for many patients with coronary artery disease [1]. Stent design has evolved through various iterations, with the most important advance being the development of drug-eluting stents (DESs). These advances were serially evaluated in randomized clinical trials, often using restenosis as an endpoint.

RCTs are widely accepted as the gold standard for the evaluation of new treatments [2]. The design, conduct, analysis, and reporting of RCTs should follow specific guidelines in order to provide valid results and avoid common pitfalls [3]. However, RCTs assessing stents face specific issues related to difficulties in blinding, the complexity of the intervention, the influence of healthcare providers, and centers' volume of care on treatment effect [4-8]. For example, there are important variations and evolutions in the techniques used for stenting, such as balloon inflation pressure and use of intravascular ultrasound guidance, as well as in the type, dosing, and duration of the pharmacological adjuvant therapy [9]. In observational studies, the magnitude of differences in outcomes related to these factors vastly exceeds those related to use of new drugs or devices [7]. The reporting of these data is therefore critical for an accurate appraisal of the risk of bias and of the applicability of the results of RCTs [10,11].

In the present study, we systematically appraised the reporting of internal and external validity data in published reports of RCTs assessing stents for percutaneous coronary interventions (PCIs).

## Methods

### Search strategy and study selection

We identified all reports of RCTs published between January 1, 2003, and September 30, 2008, that assessed stents. We searched MEDLINE using the PubMed interface and the Cochrane Central Register of Controlled Trials (issue 1, 2005) by using the terms *implantable device *OR *stents *[Mesh Terms] and *cardiovascular disease *[Mesh Terms] with a limitation to clinical trials published in English.

One author assessed the retrieved articles and screened the titles and abstracts to identify relevant studies. We included articles only if the study was identified as an RCT, was published as a full-text article, and assessed stents for PCI. We excluded case series, uncontrolled studies, articles published as abstracts only, editorials, news, correspondence sections, articles not including a complete description of the methods, and trials assessing other implantable devices (e.g., pacemaker, defibrillator, or cardiac valve) or stents in other vascular diseases. Reports of RCTs assessing technical interventions or surgical procedures where the use of stents was not systematically required were also excluded. We screened articles for duplicate publication (i.e., the same trial published with results from different lengths of follow-up), and selected only the original articles.

### Data extraction

From a review of the relevant literature and according to the CONSORT Statement guidelines [3], we generated a standardized data collection form that was iterated among the research team [5]. Before data extraction, as a calibration exercise, two members of the team (M.E., I.B.) independently evaluated a separate set of 20 reports. A meeting followed in which the ratings were reviewed and disagreements were resolved by consensus. One reviewer (M.E.) independently completed all the data extractions. A second member of the team (I.B.) reviewed a random sample of 25 articles as a quality assurance exercise. The data abstraction form is available upon request [see additional file 1].

### Trial characteristics

We collected data on trial characteristics: year of publication, funding source (public, manufacturer, or both), number of centers, setting (primary, secondary, or academic), sample size, primary and secondary outcomes, experimental treatment (DES, bare-metal stent [BMS], polymer-coated stent, specific procedure of implantation such as intravascular ultrasound-guided stenting that could involve various categories of stents), and control treatment (stent, specific procedure of implantation, surgery, angioplasty, pharmacological treatment, or other). We also checked whether statistical analyses were reported to have been performed by a center independent of the sponsor.

### Study quality

The quality of reporting was assessed using CLEAR NPT – a checklist specifically developed to evaluate the quality of RCTs assessing nonpharmacological treatments [12]. These items focus on the reporting of the generation of allocation sequence; allocation concealment; details of the intervention administered in each group; operator volume; blinding of patients, care providers and outcome assessors; follow-up schedule; and intention to treat analysis. We also assessed whether the groups were described as being similar at baseline regarding the main prognostic factors and whether eligibility criteria were specified.

### Outcomes

We checked whether the primary outcomes concerned a clinical event such as death, cardiac death, myocardial infarction, stroke, and revascularization, or an angiographic surrogate outcome such as coronary restenosis or late lumen loss.

### Description of the intervention

We recorded reporting of details on the intended interventions and on the procedural characteristics as they were actually implemented. We checked which component of the intervention was described: anesthesia management, access site, equipment (e.g., wire, guide), stent (e.g., device description, manufacturer), the procedure (e.g., use of predilatation balloon, number of inflations, duration of inflations, number of implanted stents, number of attempted lesions successfully treated, procedure duration), co-interventions and adjuvant pharmacotherapy (either mandated or left to operator's discretion).

The reporting of a method to standardize the procedure, a definition of successful procedure, and the reporting of the rate of successful procedures was also recorded.

### Description of care providers and centers

Data were recorded on the number of centers involved, center volume for the experimental treatment and for similar interventions, and the equipment in each center. We checked whether the list of centers was provided along with the number of patients treated in each. Additionally, the following data on the care provider were retrieved: reporting of selection criteria for operator (i.e., operators reported as experienced, trained, or as having performed a specific number of interventions, operators' years of practice or rates of complications); the number of operators performing the experimental intervention; and the number of patients treated by each operator.

Finally, we checked whether the clustering effect of patients by healthcare providers and centers was taken into account. In fact, in trials assessing nonpharmacological treatments, observations for participants treated by the same healthcare provider are not independent but may be clustered in individually randomized trials. This type of clustering is likely to affect the effect estimates because it will inflate the standard error and reduce the effective sample size, thus reducing the power of the trial [13,14]. This type of clustering should consequently be taken into account in sample size calculation and statistical analyses.

### Statistical analysis

We reported descriptive statistics for quantitative variables: mean, standard deviation (SD), median (Q1 to Q3), and minimum and maximum values. Categorical variables are described with frequencies and percentages. We compared the quality of reporting (i.e., number of items of CLEAR NPT adequately reported) and the sample size according to the category of stent used (active stent [drug eluting or polymer coated] versus BMS), the journal's impact factor (<3 versus = 3), and whether the report followed the CONSORT statement (reporting guidelines comprising a checklist and flow diagram to help improve the quality of reports of RCTs) in the framework of linear models with mixed effects. For instance, in a first model, the percentage of items with external validity was the dependant variable, the category of stents was the fixed effect on which F test was performed and journal was entered in the model as a random effect. So, mean comparisons of percentage of items with external validity between active and BMS stents were adjusted for the clustering effect of articles by journals as been as recommended (15).

All analyses were performed using the SAS system for Windows, release 9.1 (SAS Institute, Cary, NC).

## Results

### Selected articles

We screened the titles and abstracts of 867 potentially eligible reports; we examined the full text of 255 articles and identified 132 studies that met our inclusion criteria [See additional file 2].

The trial characteristics are reported in Table 1. Twenty (15.2%) articles were published in a general medical journal. The median sample size was 388.6 (Q1 to Q3 109.5 to 496.5) patients. The source of funding was totally or partially private in 56 (42.4%) reports and was not reported in 57 (43.2%). The statistical analyses were managed by independent centers in 26 (20.0%) reports.

**Table 1:** Reports' characteristics

	n (%) n = 132
Journal	
General medical journal	20 (15.2)
Circulation	15 (11.4)
American Heart Journal	14 (10.6)
Catheter and Cardiovascular Intervention	18 (13.6)
Journal of the American College of Cardiology	17 (12.9)
American Journal of Cardiology	15 (11.4)
Other	33 (25.0)
Funding	
Public funding	16 (12.1)
Manufacturer funding	49(37.1)
Both public and manufacturer funding	7 (5.3)
No funding	3 (2.3)
Not reported	57 (43.2)
Interventions	
BMS	41 (31.1)
Polymer-coated stent	19 (14.4)
DES	64 (48.5)
Strategy of stent implantation	8 (6.1)
Comparisons (experimental intervention vs control arm)	
DES vs BMS	35 (26.5)
DES vs another DES	19 (14.4)
DES vs same DES but with a different dosage	5 (3.8)
DES vs balloon angioplasty	6 (4.5)
DES vs polymer-coated stent	3 (2.3)
DES vs surgery	1 (0.8)
	
Polymer-coated stent vs BMS	13 (9.8)
Polymer-coated stent vs angioplasty	3 (2.3)
	
BMS vs another BMS	13 (9.8)
BMS vs angioplasty	10 (7.6)
BMS vs surgery	9 (6.8)
BMS vs a strategy of stent implantation	6 (6.8)
	
Strategy of stent implantation vs another strategy of stent implantation	4 (3.0)
Strategy of stent implantation vs angioplasty	5 (3.8)

BMS = bare-metal stent

DES = drug-eluting stent

### Reporting on center and care provider

Over half (47.7%, n = 63) of the trials were multicenter (Table 2). The median number of centers was 15.4 (Q1 to Q3 1 to 22). The number of participating centers was not reported or was unclear in 45 (34.1%) reports; the setting was described in 19 reports. The authors provided a list of participating centers in 45 (34.1%) reports. The volume of interventions performed by each center was described in only 2 (1.5%) reports.

**Table 2:** Reporting of the different components of the intervention intended or actually administered

Reporting of	n = 132 (%)
Intervention as intended	121 (91.7)
Intervention as actually administered	98 (74.2)
Component of the intervention described	
Anesthesia management	1 (0.9)
Access site (i.e. transfemoral access site)	21 (15.9)
Data on equipment (i.e., guide catheters, wires)	28 (21.2)
Data on stent	99 (75.0)
Left to operator's discretion	5 (3.8)
Description of the device (i.e., length, component)	74 (56.1)
Manufacturer	83 (62.9)
Procedural characteristics	98 (74.2)
Number of stents implanted	73 (55.3)
Use of dilatation balloon	59 (44.7)
Number of inflations	9 (6.8)
Duration of inflation	12(9.1)
Number attempted and successfully treated	12 (9.1)
Procedure duration	8 (6.1)
Co-interventions	124 (93.9)
Setting	
Secondary setting	1 (0.8)
Tertiary or academic setting	18 (13.6)
Not reported	113 (85.6)
Center	
Single	24 (18.2)
Multicentre	63 (47.7)
Not reported or unclear	45 (34.1)
Centers	
Stratification on centers	10 (7.6)
Number of centers (median, Q1 to Q3)	15.4 (1–22)
List of participating centers	45 (34.1)
Center volume reported	2 (1.5)
Source of equipment reported	1 (0.8)
Specific equipment required	0
Operators	
Selection criteria for operators	5 (3.8)
Number of operators (median, Q1 to Q3)	5.5 (5–6)
Number of patients treated by each operator	0
Clustering effect taken into account	0

Selection criteria for care providers were reported in five (3.8%) reports. These criteria were related to the participation of "experienced" care providers, with no details on the definition of "experienced". The number of care providers performing the intervention or the number of patients treated by each care provider was never reported. The clustering effect of participants by centers or by healthcare providers was never taken into account.

### Trial intervention

At least some details of the intended and actual interventions for the experimental group were available in 121 (91.7%) and 98 (74.2%) reports, respectively (Table 2). Anesthesia management was described in 1 (0.9%) report, arterial access site in 21 (15.9%) reports, and data related to the equipment used in 28 (21.2%) reports. Limited data related to the procedural characteristics were described in 98 (74.2%) reports. These data pertained mainly to the number of stents implanted and to details regarding the inflation balloon. In 49 (37.1%) reports, no information was provided on the stent manufacturer. The use of specific methods to standardize the procedure was never reported. A definition of a successful intervention was provided in 51 (38.9%) reports. The rate of successful interventions was reported in 63 (48.5%) reports. Co-interventions were described in 124 (93.9%) reports.

### Outcomes

The primary outcome relied on surrogate angiographic evaluation in almost half of the reports (Table 3). In 18 (13.6%) reports, angiography was a component of a composite outcome and in 19 (14.4%) it was a secondary outcome. Coronary angiograms were evaluated in 99 reports and were reported as standardized in 41.2% (40 of 99). Assessment of angiographic results was reported as centralized in 68.7% (68 of 99) of reports and blinded in 56.6% (56 of 99).

**Table 3:** Primary outcomes reported in randomized controlled trials assessing stents

	Primary Outcome N (%) N = 132
Angiographic evaluation (e.g., coronary restenosis)	62 (47.0)
Major cardiac events and repeat revascularization	25 (18.9)
Major cardiac events, repeat revascularization and angiographic evaluation	8 (6.1)
Repeat revascularization	7 (5.3)
Major cardiac events	8 (6.1)
Other	22 (16.7)

### Trial quality

Trial quality according to the CLEAR-NPT checklist is described in Table 4. For 8 out of 12 quality indexes in the checklist, the overwhelming majority of reports failed to provide appropriate information. The generation of allocation sequence was adequate in 31 (38.8%) reports; treatment allocation was concealed in 21 (26.3%). Patients, care providers, and outcome assessors were adequately blinded in approximately one-fifth of the reports. An intention to treat analysis was described in 56 (70.0%) reports. Patient eligibility criteria were specified in all reports.

**Table 4:** Assessment of the quality of selected randomized controlled trials using the CLEAR NPT checklist

	Yes n (%)	No n (%)	Unclear n (%)
Adequate generation of allocation of sequence	77 (58.3)	0	55(41.7)
Concealment of treatment allocation	46 (34.8)	0	86 (65.2)
Details of intervention used in each group available	125 (94.7)	0	7 (5.3)
Care providers' experience or skill in each arm appropriate	3 (2.3)	0	129 (97.7)
Participants adequately blinded	23 (17.4)	63 (47.7)	46 (34.9)
Care providers adequately blinded	16 (12.1)	74 (56.1)	42 (31.8)
If patients and/or care providers were not adequately blinded:			
All other treatments and care were the same in each group	97 (73.5)	5 (3.8)	9 (6.8)
Withdrawals and lost to follow-up were the same in each group	46 (34.8)	6 (4.5)	61 (46.2)
Outcome assessors adequately blinded to assess the primary outcomes	39 (29.5)	44 (33.3)	49 (37.1)
If outcome assessors were not adequately blinded:			
Specific methods were used to avoid ascertainment bias	2 (1.5)	13 (9.8)	76 (57.6)
Follow-up schedule was the same in each group	105 (79.5)	2 (1.5)	23 (17.4)
Main outcomes analyzed according to the intention-to-treat principle	105 (79.5)	17 (12.9)	10 (7.6)

### Factors associated with good reporting

The quality, measured by the median [Q1 to Q3] number of items on the CLEAR NPT checklist that were adequately reported, was higher for trials published in journals with a high impact factor versus those in a lower impact factor journal (4.0 [3.0 to 7.0] versus 3.0 [1.0 to 5.0]; p = 0.007) and in journals endorsing the CONSORT statement versus those not (7.0 [4.0 to 8.0] versus 4.0 [2.0 to 6.0]; p = 0.002), but was statistically different for active stent vs BMS (p < 0.0001).

The mean (SD) sample size was higher in journals with a high impact factor (469.2 [427.7] vs 251.8 [328.1]; p = 0.004) and when published in journals endorsing the CONSORT statement 750.6 [538.9] vs 335.1 [355.6]; p = 0.002), but was not statistically different for active stent vs BMS.

## Discussion

This study evaluated the reporting of the results of RCTs assessing stents for PCIs published between January 2003 and September 2008. Several studies have assessed the methodological quality of a broad range of reports of randomized trials in several areas of health care [15-17]. Concerns have been raised regarding the quality of trials assessing DESs [18]. However, to the best of our knowledge, no study has systematically assessed the quality of reporting of trials performed in this field.

Although some important data related to the description of the intervention intended and actually administered, and co-interventions provided, were adequately reported, our results highlight poor reporting of data related to the internal validity (i.e., unbiased estimates of treatment effect) and external validity (i.e., applicability of the results) of the trials.

The assessment of internal validity highlights important pitfalls: treatment allocation was concealed in only 34.8% of the reports; blinding of outcome assessors was reported in approximately one-third of the reports; and intention-to-treat analysis was reported in 79.5% of the studies. Lack of reporting of these data is associated with an increasing risk of bias, in the form of exaggerated and possibility spurious estimates of treatment effects [19].

The choice of the primary outcome in these trials also raises some concern. In about half of the reports, the main outcomes relied on angiographic evaluation such as coronary restenosis or late lumen loss. These outcomes are surrogates of clinical events and their relevance may be questionable. Marked increases in late lumen loss (>fourfold difference) are not necessarily associated with substantial differences in major cardiac events, and thus the validity of these surrogate endpoints is questioned [18,20]. Further, clinicians may extrapolate these results and consider the results of the trial equivalent to clinically relevant efficacy.

In about 20% of the reports, the main outcome was a composite associating major adverse cardiac events and revascularization. Clinical trials often use composite endpoints to reduce sample size requirements. However, such measures may prove challenging for the interpretation of results, particularly if the component endpoints are of widely differing importance to patients and the magnitude of effect differs markedly across components [21-23].

Both European Society of Cardiology [24] and American College of Cardiology/American Heart Association/Society for Cardiovascular Angiography and Interventions [24] guidelines indicate that elective PCIs should be performed by operators with acceptable annual volume at high-volume centers with on-site cardiac surgery facilities [25]. In fact, there is abundant evidence that hospitals with a larger volume of activity tend to have better outcomes and that care providers' volume of work is also a determinant for outcomes following revascularization [4,25-29]. The organization of the hospital (e.g., on-site cardiologist, activation of the catheterization laboratory by emergency physician or prehospital personnel) also impacts outcomes [30]. This is even more marked in the context of acute coronary syndromes [7,31]. Surprisingly, data related to the number and expertise of the centers and operators involved in the trial were lacking, and the potential impact of the volume was never adequately reported or taken into account in the planning (stratification) or the analyses. Consequently, readers are unable to appraise the reports adequately. In fact, an intervention might be found to be safe and effective in an RCT performed in high-volume centers by high-volume operators, but it could not be assumed that these results put into practice in low-volume centers would be identical. Unequal expertise of healthcare providers in each arm could also bias treatment-effect estimates [32]. Likewise, procedure characteristics (inflation number, duration or maximal pressure) and details on the surrounding management, such as data on equipment, access site, anaesthesia management or adjuvant therapy, were frequently lacking.

Finally, in trials assessing stents, operators are integral parts of the intervention, and observations on participants treated by the same operator may be somewhat similar or clustered [13]. This clustering will inflate standard error and reduce trial power. Furthermore, in these settings, the assumption of independence of data is violated, which means that standard statistical analyses are invalid and may give misleading conclusions. However, this issue was never addressed in the statistical analyses or the sample size calculations [14].

### Study limitations

Our search strategy and selection criteria for the reports assessed might not be comprehensive. In fact, many trials evaluating PCIs also use stents, and these were not included since stents were not the experimental therapy. However, our aim was to focus only on trials specifically assessing stents and our panel is representative of the published trials. Our analysis is based on reports of RCTs rather than on the trials themselves. Clearly, failure to report is not equivalent to failure to actually carry out the procedure or to implement adequate methods [20]. Consequently, poor or insufficient reporting is not necessarily equivalent to low quality trials. However, the published report is the only document available for readers to appraise the quality of trials, particularly in meta-analyses and systematic reviews. Empirical evidence of bias also relies mainly on the reporting of trials [19,33].

## Conclusion

This study highlights the inadequate reporting of contemporary trials involving stents. Such inadequate reporting is particularly problematic, as the technical advances tested are often rapidly implemented in clinical practice without the possibility for an adequate critical assessment of the methods used to test them.

It is desirable to increase the awareness of interventional cardiology trialists regarding checklists and guidelines for reporting trial quality such as the CONSORT Statements. With access to electronic reporting, detailed reporting of methods and quality assurance is easy to implement, and would substantially increase the quality of reporting. This would be valuable to interventional cardiologists and to the broader cardiology community for proper interpretation of the evidence regarding the use of stents in PCI.

## Abbreviations

BMS: bare-metal stent; DES: drug-eluting stent; ICC: Intraclass Coefficient Correlation; PCI: percutaneous coronary intervention; RCT: randomized controlled trial; SD: standard deviation.

## Competing interests

The authors declare that they have no competing interests.

## Authors' contributions

Conception and design: ME, IB, CR, PhGS, PhR. Acquisition of data: ME, IB, Analysis and interpretation of data: ME, IB, CR, PhGS, PhR, Drafting the manuscript: ME, IB, Final approval of the version to be published: ME, IB, CR, PhGS, PhR.

## Pre-publication history

The pre-publication history for this paper can be accessed here:

http://www.biomedcentral.com/1471-2288/9/24/prepub

## Supplementary Material

Additional File 1 **Abstraction form**. The data were recorded with an standardized abstraction form. Click here for file

Additional File 2 **The study screening process**. The data provided the study screening process. Click here for file
