# Supplementary material for: Quality of reporting internal and external validity data from randomized controlled trials evaluating stents for percutaneous coronary intervention
Source: BMC Med Res Methodol. 2009 Apr 9;9:24. doi: 10.1186/1471-2288-9-24 (PMC2679061; doi:10.1186/1471-2288-9-24)
Supplement: Additional File 1 — Abstraction form. The data were recorded with an standardized abstraction form. [file 1471-2288-9-24-S1.doc]

# Quality of Reporting Internal and External Validity Data from Randomized Controlled Trials Evaluating Stents for Percutaneous Coronary Intervention

**ARTICLE**

**Number** ………………………………………………………………………………………

**Title** …………………………………………………………………………………………

**First author** …………………………………………………………………………………

**Date of publication (year)** ………………………………………………………………… **Journal** …………………………………………………………………………………

**Impact factor of the journal** …………………………………………………………………

**Journal :**

❑ Acta Cardiol

❑ American Heart Journal

❑ American Journal of Cardiology

❑ American Journal of Medicine

❑ Annals of Internal Medicine

❑ Annals of Thoracic Surgery

❑ BMJ

❑ Cathet and Cardiovasc Intervent

❑ Circulation

❑ European Heart Journal

❑European Journal of CardioThoracic Surgery

❑ Health Technol Assess

❑ Heart Surg Form

❑ Immunology letters

❑ Int J Cardiol

❑ Int J Cardiovasc Intervent

❑ Italian Heart Journal

❑ JAMA

❑ Journal of American College of Cardiology ❑ Journal of Endovascular Therapy

❑Journal of Invasive Cardiology

❑ Journal of Nephrology

❑ Journal of Neurosurgery

❑Journal of Vascular Intervention Radiol

❑ Journal of Vascular Surgery

❑Lancet

❑ Medicine

❑ New England Journal of Medicine.

❑ Neurosurgery

❑ Semin Vasc Surg

❑ Stroke

❑ Transplantation Proceeding

❑ Other

**DEMOGRAPHIC DATA**

**Funding Yes No**

- Public funding reported
- Manufacturer support reported
- Funding source not reported or unclear

Were data reported has managed by an independent center

(different to the sponsor)

**Number of patients randomized in the trial** |__|__|__|__|__|

**Number of centers** |__|__|__|  unclear multicentric  unclear

Yes No Not Reported

- Was the trial stratified on the center

**Setting**

- - I
  - II
  - III/Academic
  - Not reported
  - Other

**Yes No**

**Was centers’volume reported?**

If yes, how ?

- Interventions
  - Studied intervention
  - Similar intervention
- Other

**Equipement :**

**Were data on the source of equipement of the center reported ?**

**Were specific equipement required in all centers ?**

**Was a list of center provided?**

# interventions

**Medical device (FDA definition):** product which does not meet the definitions of a drug and is essentially any artifact, article or tool intented to affect the structure or the function of humans or animals

**Number of arm** |__|__|

1. **Experimental intervention**

**Yes No**

- Coated stent
- Eluting stent (coated stent+drug)
- Biodegradable stent
- Metallic stent / control bare stent /

bare metal stent / stainless stent

- A specific procedure of stent implantation
- A strategy of stent implantation

**Yes No Not reported**

Were all co-intervention left to care provider discretion?

**IF no,**

Was a co-intervention systematically administrated?

**2. Control arm 1**

**Yes No**

- Same eluting stent but with a different dosage
- Coated stent
- Eluting stent (coated stent+drug)
- Biodegradable stent
- Metallic stent / control bare stent /

bare metal stent / stainless stent

- Another specific procedure of stent implantation
- Surgery
- Drug alone
- Usual care
- Other .................................
- Angioplasty

**Yes No Not reported**

Were all co-intervention left to care provider discretion?

**IF no,**

Was a co-intervention systematically administrated?

**Control arm 2 [ IF MORE THAN 2 ARM]**

**Yes No**

- Same eluting stent but with a different dosage
- Coated stent
- Eluting stent (coated stent+drug)
- Biodegradable stent
- Metallic stent / control bare stent /

bare metal stent / stainless stent

- Another specific procedure of stent implantation
- Surgery
- Drug alone
- Other .................................

**Yes No Not reported**

Were all co-intervention left to care provider discretion?

**IF no,**

Was a co-intervention systematically administrated?

# intervention

**Yes No**

1. Were details of the interventions **intended** for the intervention

group reported?

If **YES**, how was the procedure described?

- usual care (left to care provider discretion)

- described in the text or table

- only inventoried in the text

- other

**Did a description concern?**

- anaesthesic management

- access site

- data on equipement (guide catheters, wires)

- Data on stent

- Left to care provider discretion
- device description (matériau, longueur)
- manufacturer

- use of dilatation ballon

- nursing care

1. Were details of the **real** interventions for each group described?

If **YES**,

- described in the text or table

- other

**Did a description concern?**

- anaesthesic management

- access site

- manufacturer

- device description (longueur, matériau)

- number of inflation

- duration of inflation

- number of implanted stent (median, %)

- number of attempted lesions successfully treated

- procedure duration

1. Was the method to standardized the procedure reported?
2. Was a definition of successfull intervention provided?
3. Was the rate of successfull intervention reported?

# care provider

**Data on care providers : Experimental intervention**

|  | **YES** | **NO** |
| --- | --- | --- |
| - - **Were selection criteria (years of practice etc…) of the care providers reported?** |  |  |
| **IF YES,** |  |  |
| - Care providers were reported as “experienced” with no details reported? |  |  |
| - Were data on Years of practice reported? |  |  |
| - Were data on number of experimental interventions performed reported? |  |  |
| - Were data on care providers’ skill assessed by level of complication when performing the experimental intervention reported? |  |  |
| - Were data on care providers’ skill on video reviewed by an executive committee reported? |  |  |
| - Were data on specific training of care providers reported? |  |  |
| - Other………………… |  |  |
|  | **YES** | **NO** |
| - - **Was the number of care providers performing the experimental intervention reported?**   **IF YES N=** |__|__|__| |  |  |
| - - **Was the number of patients treated by each care providers in the experimental arm reported?** |  |  |
| - - **Were details of important surrounding care providers reported in the experimental arm?** |  |  |
| - **Other** |  |  |

# TRIAL QUALITY EVALUATION

# CLEAR : Final checklist of items to assess quality of randomized controlled trials of nonpharmacological treatment.

1. **Was the generation of allocation sequences adequate?** Yes / No / Unclear
2. **Was the treatment allocation concealed?** Yes / No / Unclear
3. **Were details of the intervention administered to each group made available[[1]](#footnote-2)?** Yes / No / Unclear
4. Were care providers’ experience or skill*[[2]](#footnote-3)* in each arm appropriate*[[3]](#footnote-4)*? Yes / No / Unclear
5. **Were participants adequately blinded?** Yes /

No, because blinding is not feasible /

No, although blinding is feasible /

Unclear

- 1. **If participants were not adequately blinded**
     1. **Were all other treatments and care (ie, co-interventions) the same in each randomized group?** Yes / No / Unclear
     2. **Were withdrawals and lost to follow-up the same in each randomized group?** Yes / No / Unclear

1. **Were care providers or persons caring for the participants[[4]](#footnote-5) adequately blinded?** Yes /

No, because blinding is not feasible /

No, although blinding is feasible /

Unclear

- 1. **If care providers were not adequately blinded**
     1. **Were all other treatments and care (ie, co-interventions) the same in each randomized group?** Yes / No / Unclear
     2. **Were withdrawals and lost to follow-up the same in each randomized group?** Yes / No / Unclear

1. **Were outcome assessors adequately blinded to assess the primary outcomes?** Yes /

No, because blinding is not feasible /

No, although blinding is feasible /

Unclear

- 1. **If outcome assessors were not adequately blinded, were specific methods used to avoid ascertainment bias (systematic differences in outcome assessment)[[5]](#footnote-6)**  Yes / No / Unclear

1. **Was the follow-up schedule the same in each group[[6]](#footnote-7)?** Yes / No / Unclear
2. **Were the main outcomes analyzed according to the intention-to-treat principle?** Yes / No / Unclear

# EVALUATION : MACES

Primary Secondary No

outcome outcome

- - Death (all causes)
  - Cardiac death
  - Myocardial infarction
  - Stroke
  - Revascularisation
    - **If yes**, was a systematic complementary test

performed to detect vascular stenosis reported  (yes)  (no)

- - Structural outcome

(endovascular echography, arteriography)

1. Was the acquisition of structural outcomes

- standardized
- blinded

1. Was the assessment of structural outcome reporting (reading)

- Centralized
- blinded
  - Need for drug (example : antianginadrug)
  - Reccurrence of angina
- Other : ………………………

# ADVERSE EVENTS

1. **CLARIFY HOW HARMS–RELATED INFORMATION WAS COLLECTED**

Yes No

1) **Was the nature of adverse events collected reported?**

IF YES

a) **Did these events concern only MACE or MACCE?**

1. **Were mode of data collection reported?**

**If yes**, which method was used?

- - - Questionnaires (questions fermées)
    - Interview (questions semi-dirigées)
    - Specific standardised examination (for search AE)
    - Predetermined complementary examination
    - Other

a) **Did these methods concern only MACE or MACCE?**

3) **Were time frame of surveillance for adverse events specified?**

2) **BLINDING**

**Yes No Not reported**

- Was the assessment of at least one adverse event blinded?

- Was the assessment of adverse event severity blinded?

a) Did blinding concern only MACE or MACCE?

1. **REPORTING OF AE**

**Yes No**

1. **Was it stated that no AE occured?**
   1. **if yes,**

- was the nature of AE monitored reported

- 1. **If no**,

Were adverse events reported?

*If YES*, how

- Numerical data
- Generic statement
- Globally
- Per arm
- In only one arm

**Did these data concern only MACE or MACCE?**

**Yes No**

**Were AE nature reported?**

**IF YES**

How?

- Globally

- Per arm
- In only one arm

**Did these data concern only MACE or MACCE?**

**Were AE severity reported?** (minor, severe, death)

**IF YES**

How?

- generic statement

- numerical data

- Globally
- Per arm
- In only one arm

**Did these data concern only MACE or MACCE?**

**Was a definition of expected AE reported?**

**Was a grading of expected AE reported ?**

**Yes No Not reported**

**Was the period after the procedure for reporting**

**adverse events reported ?**

**If yes,**

- Less than one month follow up
- 1 month follow up
- 1-5 months follow up
- 6 months follow up
- 7-11 months follow up
- 12 months follow up
- More than one year follow up

**Did these data concern only MACE or MACCE?**

**SPACE ALLOCATED TO ADVERSE EVENTS**

- Space allocated to adverse events reported in results part: |__|__|__| lignes

- Space allocated to results part : |__|__|__| lignes

- Number of tables allocated to adverse events…………………..|__|__|__| tables
- Number of figures allocated to adverse events…………….. |__|__|__| figures
- Number of tables …………………..|__|__|__| tables
- Number of figures …………….. |__|__|__| figures

**ATTRIBUTION** (process of deciding wether an adverse event is related to the intervention)

**Yes No**

Were the adverse events related to the treatment reported?

**IF YES, how**

- Generic statement
- Numerical data

**If yes**, imputability of adverse events was reported:

- - Globally
  - Per arm
  - In only one arm
- Was a specific tool used for adverse events imputability   reported?

IF YES, Which one? .....................................

- Were procedural complications reported?

**Yes No**

Was the causality of AE reported ?

If **yes,** was it linked to ?

- **Device effect**
  - Presence of the device (dissection, ↑ CPK,

↑ troponine, thrombose)

- - Device failure
  - Complications related to associated drug
  - Other
- **Co-associated treatment**
- **Gesture effect**
  - Implantation procedure
  - Other
- **Idiosyncratic patient reactions (allergy)**
- **Other**

- - 1. **ADJUDICATION COMMITTEE**

**Yes Not reported**

- Were events assessed by an adjudication committee?

**If yes,**

- Was the adjudication committee blinded?
  - Was a description of members reported?

**If only part** of events was assessed by the adjudication

committee, was a specific rule for submitting adverse events

to the committee reported?

**G. DATA SAFETY MONITORING COMMITTEE (DSMC)**

**Yes Not reported**

- Was there a DSMC?

**If yes,**

- Were adverse effects assessed by a DSMC ?
- Was a description of members reported?
- Was the DSMC assessed ?
  - All adverse events
  - Only part of adverse events
  - Only the adverse events assessed by the adjudication committee
  - All the severe or serious AE
  - All the AE related to the device

**Commentaries**

…………………………………………………………………………………………………

……………………………………………………………………………………………………………………………………………………………………………………………………

…………………………………………………………………………………………………

……………………………………………………………………………………………………………………………………………………………………………………………………

…………………………………………………………………………………………………

1. The answer should be “**yes**” for this item if these data were either described in the report or made available for each arm (reference to a preliminary report, online addendum etc.) [↑](#footnote-ref-2)
2. Care providers’ experience or skill will be assessed only **for** **therapist-dependent interventions** (i.e., interventions where the success of the treatment are directly linked to care providers’ technical skill and where care providers are part of the treatment procedure). For other treatment, this item is **not relevant** and should **be removed** from the checklist or answered “unclear”. [↑](#footnote-ref-3)
3. Appropriate experience or skill is not necessarily similar in each study arm, particularly when the interventions compared are different (e.g., surgery versus rehabilitation) and making judgments about appropriate experience or skill may be difficult. Appropriate experience or skill should be determined according to published data, preliminary studies, guidelines, run-in period or, if these data are lacking, a group of experts. Appropriate experience or skill will have to be determined and prespecified in the protocol for each study arm before the beginning of the survey. [↑](#footnote-ref-4)
4. if persons administering cointerventions are blinded whereas care providers are not blinded but are not responsible for administering co-interventions answer “**yes**” [↑](#footnote-ref-5)
5. The answer should be “yes” for this item, if the main outcome is **objective or “hard**” or outcomes were assessed by a **blinded or at least an independent endpoint review committee** or outcomes were assessed by an **independent outcome assessor trained** to perform the measurements in a standardized manner or the outcome assessor was **blinded to the study purpose and hypothesis**. [↑](#footnote-ref-6)
6. This item is not relevant for trials in which **follow-up is part of the question**. For example, this item is not relevant for a trial assessing frequent versus less frequent follow-up for cancer recurrence. In these situations, this item should be **removed** from the checklist or answered “unclear”. [↑](#footnote-ref-7)
