# Supplementary material for: Quality of reporting internal and external validity data from randomized controlled trials evaluating stents for percutaneous coronary intervention
Source: BMC Med Res Methodol. 2009 Apr 9;9:24. doi: 10.1186/1471-2288-9-24 (PMC2679061; doi:10.1186/1471-2288-9-24)
Supplement: Additional File 2 — The study screening process. The data provided the study screening process. [file 1471-2288-9-24-S2.doc]

Medline and Cochrane database search

**867 reports**

Articles excluded on the basis of the title and abstract (n = 612)

Nonrandomized trials (n = 133)

Trials not assessing a stent

Not assessing a medical device (n = 365)

Defibrillator (n = 61)

Pacemaker (n = 8)

Cardiac valve (n = 6)

Subgroup analysis (n = 3)

Follow-up trial (n = 34)

Other (n = 2)

Articles selected

(n = 255)

Articles assessed (n = 132)

Bare-metal stent (n = 69)

Polymer-coated stent (n = 16)

Drug-eluting stent (n = 38)

Strategy of stent implantation (n = 9)

Articles excluded after obtaining the full text (n = 123)

Not randomized (n = 34)

Not assessing a medical device (n = 12)

Stenting not mandatory (n = 18)

Material and methods published previously (n = 16)

Meta-analysis (n = 2)

Follow-up trials (n = 16)

Subgroup analysis (n = 14)

Not assessed stent in cardiovascular disease (n = 11)
